# Carbapenem-resistant enterobacterales in sterile body fluids: ten-year population genomics and clinical risk factors in a tertiary hospital, 2016-2025

**DOI:** 10.3389/fcimb.2026.1821740

**Published:** 2026-06-02

**Authors:** Shenyun Cao, Peng Wang, Yonghua Liu, Lijuan Qi, Xinying Wang, Lin Li, Zhijun Zhang

**Affiliations:** 1 Department of Laboratory Medicine, The Affiliated Taian City Central Hospital of Qingdao University, Taian, China; 2 Shandong Provincial Key Medical and Health Laboratory of Anti-Drug Resistant Drug Research, The Affiliated Taian City Central Hospital of Qingdao University, Taian, China; 3 Department of Laboratory Medicine, Jinan Nanshan People’s Hospital, Jinan, China; 4 Department of Laboratory Medicine, Binhai New Area Hospital of Traditional Chinese Medicine (TCM), Tianjin, China; 5 Department of Hematology, The Affiliated Taian City Central Hospital of Qingdao University, Taian, China; 6 Pharmacy Intravenous Admixture Services, The Affiliated Taian City Central Hospital of Qingdao University, Taian, China

**Keywords:** carbapenem-resistant enterobacterales, hypervirulence, rmpA2, ST11, sterile body fluids

## Abstract

**Objectives:**

To clarify the molecular epidemiology, resistance gene profiles, virulence characteristics, and clinical prognosis-related factors of Carbapenem-Resistant Enterobacterales (CRE) isolated from sterile body fluids. This study provides microbiological evidence for clinical management and infection control surveillance.

**Methods:**

62 non-duplicate sterile fluid CRE strains from 2016–2025 were retrospectively analyzed. VITEK-2 Compact detected MICs per CLSI M100. Illumina NovaSeq whole-genome sequencing was assembled via ABySS and GapCloser. Databases analyzed resistance, virulence, plasmid and MLST profiles. SNP phylogenetic analysis identified clonal clusters and strain genetic relationships.

**Results:**

Among the 62 CRE isolates, Klebsiella pneumoniae was the predominant species (77.4%, 48/62), followed by Escherichia coli (12.9%, 8/62) and Enterobacter cloacae complex (6.5%, 4/62). Additionally, single isolates of Klebsiella aerogenes and Citrobacter freundii were recovered. A total of 6 sequence types (STs) were identified, with ST11 being the most prevalent (87.5%, 42/48) among K. pneumoniae isolates. Carbapenemase genes were detected in 91.7% (44/48) of K. pneumoniae strains, with blaKPC-2 (85.4%, 41/48) and blaNDM-1 (10.4%, 5/48) as the main types. Three strains co-harbored blaKPC-2 and blaNDM-1, and one strain carried blaNDM-5 alone. These strains also carried class C β-lactamase (AmpC), extended-spectrum β-lactamases (ESBLs), and aminoglycoside/quinolone resistance genes. The rmpA2 virulence gene was detected in 75.0% (36/48) of K. pneumoniae isolates. Among E. coli, blaNDM-5 (4/8) and blaNDM-13 (2/8) were predominant. One ST155 isolate exhibited ertapenem resistance potentially mediated by AmpC promoter mutations and porin loss based on genomic prediction. Additionally, mcr-1.1 was detected in one ST361 isolate. All four E. cloacae complex belonged to ST171 and harboured blaNDM-1; one co-carried mcr-9.1. Clonal analysis revealed five major clusters within ST11 (Clade A-E), with Clade B comprising genetically related dual-carbapenemase isolates from multiple wards (8-10 SNPs). Twelve patients (19.4%) died. Intensive Care Unit (ICU) admission was significantly associated with mortality (83.3% vs. 30.0%, P=0.002).

**Conclusions:**

CRE from sterile body fluids were dominated by ST11 K. pneumoniae carrying blaKPC-2, with complex multidrug resistance and frequent carriage of rmpA2 and other virulence-associated genes. Continuous molecular surveillance of dominant clones and monitoring of high-risk patient populations may inform strategies to reduce CRE burden.

## 1 Introduction

Carbapenem antibiotics represent the last line of defense against infections caused by multidrug-resistant Enterobacterales. The prevalence and dissemination of Carbapenem-Resistant Enterobacterales (CRE) have emerged as a significant public health challenge in the global clinical anti-infective field (Jesudason, 2024; Perez and Bonomo, 2019). According to WHO statistics, CRE is classified as a critical priority on the priority list of human bacterial antimicrobial resistance, with infection-associated 30-day mortality rates reaching 30%-50% (Paniagua-Garcia et al., 2024). Sterile body fluid infections (including ascites, pleural effusion, cerebrospinal fluid, bile, etc.) constitute an important category of severe clinical infections. Once CRE infection occurs, the disease progresses rapidly, therapeutic options become extremely limited, and prognosis is poor (Falcone et al., 2023). Unlike respiratory tract infections or urinary tract infections, CRE infections in sterile body fluids often simultaneously reflect invasive infections and nosocomial dissemination, carrying higher mortality risks and greater urgency for prevention and control (Tamma et al., 2022; Wang et al., 2022).

In clinical practice, *Klebsiella pneumoniae* represents the most prevalent CRE isolate, followed by *Escherichia coli*, *Enterobacter cloacae complex*, and other Enterobacterales species (Chen et al., 2021). Significant differences exist among different CRE species regarding carbapenemase types, virulence characteristics, and clinical pathogenicity (Adelman et al., 2022): *K. pneumoniae* predominantly produces KPC-type enzymes. Particularly in China, the ST11-KL64/KL47 clonal groups carrying the *bla*_KPC-2_ gene have become the dominant epidemic strains and frequently fuse with hypervirulence features (such as *rmpA/rmpA2*, aerobactin gene cluster *iuc*, etc.), forming hypervirulent carbapenem-resistant *Klebsiella pneumoniae* (hv-CRKP) (Ernst et al., 2020; Pu et al., 2023; Bonomo et al., 2018). This genetic architecture differs markedly from that in North America and Europe, where ST258/ST512-KPC clones predominate (Ma et al., 2023; Mathers et al., 2015; Castanheira et al., 2013), as well as from countries such as Italy, where ST512 is specifically linked to KPC-3 dissemination (Arcari et al., 2023). *E. coli* more commonly produces NDM-type metallo-β-lactamases, with clonal groups ST131 and ST167 being globally widespread (Li et al., 2024; Alba et al., 2021). *Enterobacter cloacae complex*, as an important nosocomial pathogen, predominantly produces NDM-type metallo-β-lactamases, with high-risk clones ST171 and ST78 circulating globally (Gomez-Simmonds et al., 2018; Lumbreras-Iglesias et al., 2023). Additionally, carbapenem resistance mechanisms include chromosomal class C β-lactamase (AmpC) enzyme (ACT-16, etc.) overexpression combined with outer membrane porin deficiency, as well as the coexistence of acquired carbapenemases (KPC, OXA-48-like, etc.) with AmpC enzymes (Guerin et al., 2015). However, existing studies have largely focused on single-species CRE characteristics or have included specimens from non-sterile sites such as the respiratory tract, urinary tract, and wounds. Systematic comparative studies targeting sterile body fluids-particularly within the distinct Chinese molecular epidemiological context characterized by ST11-KPC-2 predominance and hv-CRKP emergence-remain scarce, and multi-species genomic investigations based on whole-genome sequencing(WGS) are urgently needed.

We characterized the molecular epidemiology, resistance mechanisms, and clonal transmission dynamics of CRE isolated from sterile body fluids, providing genomic evidence for clinical management and infection control surveillance.

## 2 Materials and methods

### 2.1 Specimen sources and identification of bacterial isolates

A total of 62 non-duplicated CRE strains were collected from sterile body fluids (excluding blood) at the Affiliated Taian City Central Hospital of Qingdao University between January 2016 and March 2025. Non-duplicated was defined as a single isolate per patient per species, with repeated isolates of the same species from the same patient excluded. Isolates were identified using the Autof ms 1000 automated microbial mass spectrometry detection system. The criteria for carbapenem resistance was defined as ertapenem MIC ≥ 2 mg/L or imipenem/meropenem MIC ≥ 4 mg/L (Clinical and Laboratory Standards Institute, 2024). After verification, pure cultures were suspended in brain–heart infusion broth containing 15% (v/v) glycerol and stored at −80 °C until further identification.

### 2.2 Antimicrobial susceptibility testing

Antimicrobial susceptibility testing for all strains was conducted using VITEK 2 N335 and XN04 cards. Interpretation of susceptibility results followed the Clinical and Laboratory Standards Institute (CLSI) M100-S34 guidelines (Clinical and Laboratory Standards Institute, 2024). For tigecycline, susceptibility interpretation was based on FDA-identified interpretive criteria (U.S. Food and Drug Administration, 2023), while resistance breakpoints for polymyxin B were referenced according to the European Committee on Antimicrobial Susceptibility Testing (EUCAST) standards (The European Committee on Antimicrobial Susceptibility Testing, 2024).The quality control strain was *Escherichia coli* ATCC 25922.

### 2.3 Whole-genome sequencing and assembly

Fresh CRE colonies from blood agar plates were selected, and DNA was extracted using the Bacterial DNA Kit (OMEGA) according to the manufacturer’s instructions. DNA samples were sent to Shanghai Biozeron Biological Technology Co., Ltd. for WGS. Based on the Illumina NovaSeq sequencing platform, full library construction, amplification sequencing, data processing, and quality assessment were performed for all 62 CRE strains. Genome assembly was conducted using ABySS (http://www.bcgsc.ca/platform/bioinfo/software/abyss) with multiple-k-mer parameters. Subsequently, GapCloser software (https://sourceforge.net/projects/soapdenovo2/files/GapCloser/) was adopted to fill remaining local internal gaps and correct base sequencing errors in the final assembly. The raw sequencing reads and genome assemblies corresponding to individual BioSample accession numbers are listed in **Supplementary Table 1**.

### 2.4 Detection of resistance genes, plasmids, and virulence genes

Multilocus sequence typing (MLST) and resistance gene identification (ResFinder 4.2) analyses for the 62 CRE strains were performed using the Center for Genomic Epidemiology (CGE) website (http://www.genomicepidemiology.org/services/). The VFanalyzer tool from the Virulence Factors Database (VFDB) developed by the Chinese Academy of Medical Sciences (http://www.mgc.ac.cn/VFs/) was applied to screen virulence factors in 48 CRKP strains. The VFanalyzer tool from VFDB (http://www.mgc.ac.cn/VFs/) was used to screen virulence genes in 48 CRKP strains, with the following genes being detected (Russo and Marr, 2019): *rmpA*, *rmpA2*, *fimH*, *mrkD*, *entB*, *ybtA*, *iucA*, *wzi*, *hcp/tssD*, *acrA*, and *rcsA*. Plasmid replicon analysis of 48 K*. pneumoniae* strains was conducted using PlasmidFinder from the CGE website.

### 2.5 Molecular typing and phylogenetic analysis

Multilocus sequence typing (MLST) was applied to assign sequence types (ST) by uploading the sequencing data of 62 CRE strains to the online database, with ST types determined through sequencing of seven housekeeping genes. Single nucleotide polymorphisms (SNPs) were only performed for the 48 CRKP isolates among the 62 CRE strains, analyze using the CSI Phylogeny tool from the CGE website. Clonal relatedness was defined as ≤21 SNP differences based on core genome alignment, consistent with established criteria for detecting nosocomial transmission of ST11-KL64 *K. pneumoniae* (Wang et al., 2023). Isolates differing by ≤21 SNPs were considered to belong to the same clonal cluster. Phylogenetic trees were generated based on the analysis results of 48 CRKP strains, and the evolutionary trees were visualized using the ChiPlot online platform for phylogenetic relationship analysis (Xie et al., 2023).

### 2.6 Data statistical analysis

Descriptive statistics were used to summarize clinical characteristics and microbial features. Categorical variables were expressed as counts and percentages, and continuous variables as median. Comparisons between survivors (n=50) and non-survivors (n=12) were performed using Fisher’s exact test for categorical variables and Mann-Whitney U test for continuous variables. A two-tailed P-value <0.05 was considered statistically significant. All statistical analyses were conducted using Statistical Package for the Social Sciences (SPSS) version 26.0.

## 3 Results

### 3.1 Clinical characteristics

A total of 62 CRE cases were collected between January 2016 and December 2025. The cohort comprised 41 males (66.2%) and 21 females (33.8%), with ages ranging from 0 to 96 years (median, 70 years; IQR, 60-76 years); 46 patients (74.2%) were >60 years old. The majority of patients were admitted to the intensive care unit (ICU) (25/62, 40.3%), followed by surgical departments (20/62, 32.3%, including 8 cases from gastrointestinal surgery, 4 cases from hepatobiliary surgery, 3 cases from abdominal wall and hernia surgery, etc.) and internal medicine departments (17/62, 27.4%). Thirty-six patients (58.1%) had a history of ICU admission within the past 12 months, and 48 patients (77.4%) had undergone surgical procedures within the past 6 months. Invasive procedures were common, with 47 patients (75.8%) receiving indwelling urinary catheterization, central venous catheterization, or mechanical ventilation, including 41 cases (66.1%) with urinary catheters, 34 cases (54.8%) with central venous catheters, and 24 cases (38.7%) with mechanical ventilation. Thirty-three patients (53.2%) had prior exposure to carbapenems before CRE detection. Twelve patients (19.4%) died, including 10 cases of CRKP infection, and 1 case each of *E. coli* and *E. cloacae* infection (**Table 1**). The primary specimen sources were ascites in 31 cases (50.0%), pleural fluid in 12 cases (19.4%), bile in 12 cases (19.4%), and other sources in 7 cases (11.3%). Among the 12 fatal cases, the primary specimen sources were ascites (n=6, 50.0%), pleural fluid (n=5, 41.7%), and bile (n=1, 8.3%) (**Supplementary Table 2**).

**Table 1:** Univariate analysis of factors influencing in-hospital mortality in patients with CRE sterile body fluid infection (n=62).

Variable	Survivors (%, n=50)	Non-survivors (%, n=12)	P value
Demographics			
Age, (median)	75	66.5	0.196
Sex (Male vs Female)			0.735
Female, n (%)	36.0 (18)	25.0 (3)	
Male sex, n (%)	64.0 (32)	75.0 (9)	
Clinical factors			
ICU admission, n (%)	30.0 (15)	83.3 (10)	**0.002**
Surgical history, n (%)	84.0 (42)	50.0 (6)	**0.02**
Invasive procedures, n (%)	72.0 (36)	100.0 (12)	0.052
Prior carbapenem exposure, n (%)	52.0 (26)	58.3 (7)	0.756
CRKP (vs. other species), n (%)	76.0 (38)	83.3 (10)	0.717

Bold values indicate statistically significant differences (P < 0.05).

### 3.2 Species identification and sequence typing

MALDI-TOF identification revealed that carbapenem-resistant *Klebsiella pneumoniae* (CRKP) was the predominant species (48/62, 77.4%), followed by *Escherichia coli* (8/62, 12.9%) and *Enterobacter cloacae* (4/62, 6.5%), with one case each of *Klebsiella aerogenes* and *Citrobacter freundii* (3.2%). Among the 48 CRKP strains, six ST types were identified, with ST11 being the most common sequence type (42/48, 87.5%), followed by ST1306, ST20, ST7, ST3948, and ST14 (1/48 each, 2.1%). One strain (C8) failed to obtain an ST typing result. Seven ST types were detected among the 8 Carbapenem-Resistant *Escherichia coli* (CRECO) strains, showing high genetic diversity without a predominant lineage. The ST types were distributed as follows: ST155, ST156, ST361, ST744, ST10, ST648, and ST2325 (1/8 each, 12.5%). One strain (C66) failed to obtain an ST typing result. All four Carbapenem-Resistant *Enterobacter cloacae* (CRECL) strains belonged to ST171.

### 3.3 Resistance genes and phenotypes

**Figure 1:**
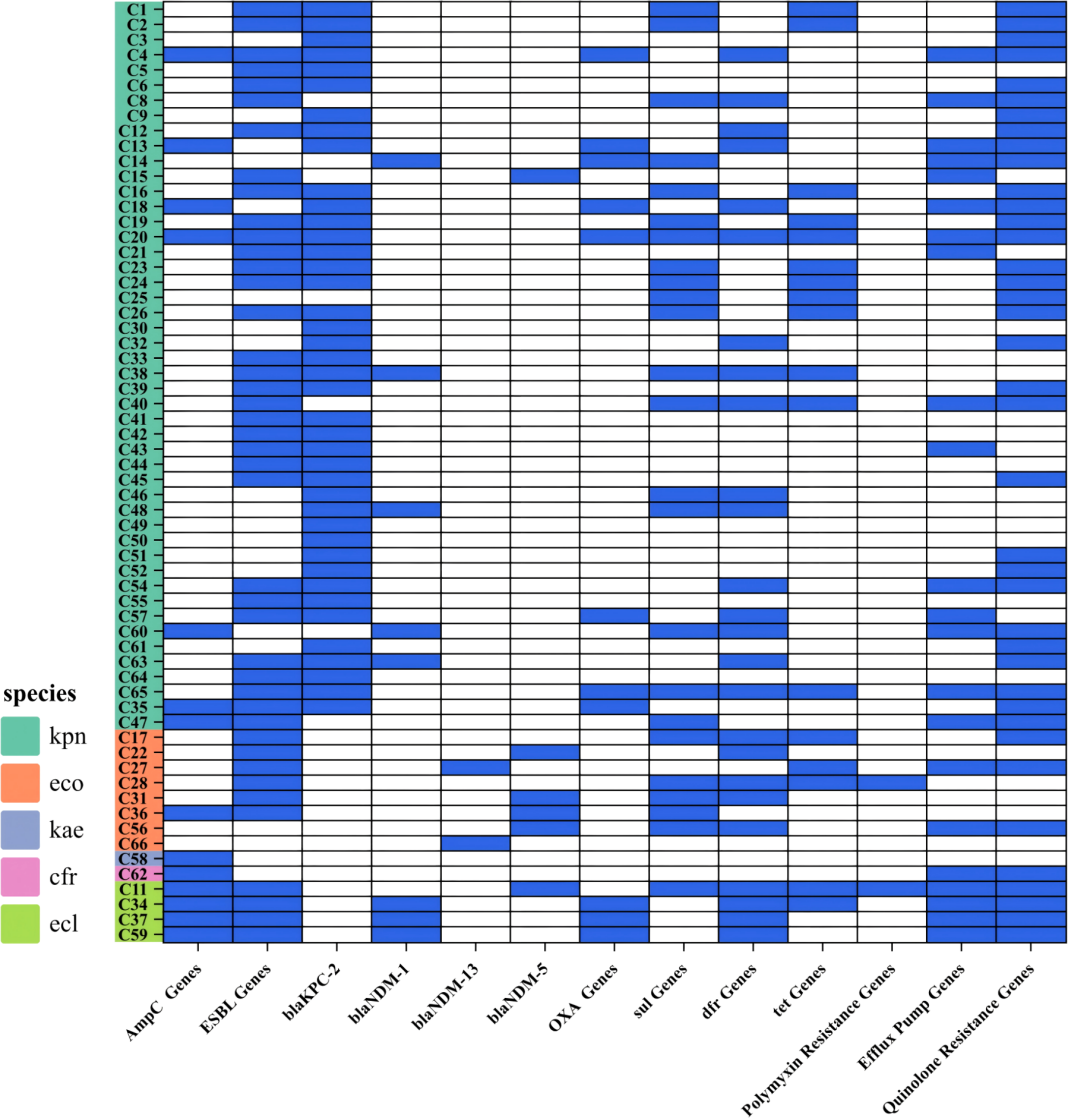
Heat-map displaying resistance-gene content of 62 CRE isolates. X-axis represents resistance genes, Y-axis shows the strain sequence numbers, Blue fill indicates presence of the corresponding gene; White fill indicates absence. Species are color-coded on the left: green, *K. pneumoniae*; orange, *E. coli*; purple, *K. aerogenes*; pink, C. freundii; light green, *E. cloacae*.

All 62 strains were multidrug-resistant, with significant interspecies differences in resistance gene carriage rates. ESBL genes were the predominant resistance genes, with an overall positive rate of 67.7% (42/62); specifically, *E. cloacae* showed 100% positivity, while *K. pneumoniae* showed 66.7% (32/48). The overall carbapenemase gene carriage rate was 87.1% (54/62), with *bla*_KPC-2_ being the most prevalent (66.1%, 41/62) and exclusively detected in *K. pneumoniae*. The positive rates for *bla*_NDM-1_, *bla*_NDM-5_, and *bla*_NDM-13_ were 12.9% (8/62), 9.7% (6/62), and 3.2% (2/62), respectively. The overall positive rates for fluoroquinolone and aminoglycoside resistance genes were 62.9% (39/62) and 56.5% (35/62), respectively. The co-carriage rate of *sul* and *dfr* genes was 21.0% (13/62). The overall positive rate for polymyxin resistance genes was only 3.2% (2/62). Additionally, *E. cloacae* exhibited 100% positivity for both AmpC and ESBL genes, with universal positivity for efflux pump and aminoglycoside resistance genes (**Figure 1**).

Antimicrobial susceptibility results: All 62 strains showed 100% resistance to cephalosporins, piperacillin/tazobactam, cefoperazone/sulbactam, and ertapenem. Resistance rates were as follows: aztreonam 95.2%, ciprofloxacin 91.9%, levofloxacin 90.3%, amikacin 43.5%, and trimethoprim-sulfamethoxazole 56.5%. Resistance rates to tigecycline, polymyxin B, and ceftazidime-avibactam (excluding NDM-producing strains) were below 5% (**Table 2**).

**Table 2:** MICs and ST types of 62 CRE isolates against 16 antimicrobial agents.

Isolation ID	Strain	Carbapenemase	ST	MIC(mg/L)															
				SCF	CRO	CAZ	FEP	ATM	ETP	IPM	MEM	TZP	LEV	CIP	AK	SXT	COL	TGC	CZA
C1	*kpn*	KPC-2	11	≥64	≥32	≥16	≥32	≥16	≥2	≥16	≥16	≥128/4	≥8	≥4	≥64	≤1/19	≤1	2	4
C2	*kpn*	KPC-2	11	≥64	≥32	≥16	≥32	≥16	≥2	≥16	≥16	≥128/4	≥8	≥4	≤2	>4/76	≤0.5	4	2
C3	*kpn*	KPC-2	11	≥64	≥32	≥16	≥32	≥16	≥2	≥16	≥16	≥128/4	≥8	≥4	≤2	2/38	≤0.5	4	2
C4	*kpn*	KPC-2	11	≥64	≥32	≥16	≥32	≥16	≥2	≥16	≥16	≥128/4	≥8	≥4	≥64	>4/76	1.5	≤2	2
C5	*kpn*	KPC-2	11	≥64	≥32	≥16	≥32	≥16	≥2	≥16	≥16	≥128/4	≥8	≥4	≥64	≤1/19	≤0.5	≤0.5	4
C6	*kpn*	KPC-2	11	≥64	≥32	≥16	≥32	≥16	≥2	≥16	≥16	≥128/4	≥8	≥4	>32	2/38	2	1	4
C8	*kpn*	ND	ND	≥64	≥32	≥16	≥32	≥16	≥2	2	≥16	≥128/4	≥8	≥4	2	>4/76	1	≤2	0.5
C9	*kpn*	KPC-2	11	≥64	≥32	≥16	≥32	≥16	≥2	≥16	≥16	≥128/4	≥8	≥4	≤2	>4/76	≤0.5	4	2
C11	*ecl*	NDM-5	171	≥64	≥32	≥16	≥32	≥16	≥2	≥16	≥16	≥128/4	≥8	≥4	8	>4/76	1.5	≤2	–
C12	*kpn*	KPC-2	11	≥64	≥32	≥16	≥32	≥16	≥2	≥16	≥16	≥128/4	≥8	≥4	0.5	>4/76	4	≤2	2
C13	*kpn*	KPC-2	11	≥64	≥32	≥16	≥32	≥16	≥2	≥16	≥16	≥128/4	≥8	≥4	≥64	>4/76	1.5	≤2	2
C14	*kpn*	NDM-1	1306	≥64	≥32	≥16	≥32	≤4	≥2	≥16	≥16	≥128/4	0.5	2	0.5	2/38	4	≤2	–
C15	*kpn*	NDM-5	20	≥64	≥32	≥16	≥32	≥16	≥2	≥16	≥16	≥128/4	0.5	0.5	0.5	>4/76	2	2	–
C16	*kpn*	KPC-2	11	≥64	≥32	≥16	≥32	≥16	≥2	≥16	≥16	≥128/4	≥8	≥4	≤2	>4/76	≤0.5	4	1
C17	*eco*	ND	155	≥64	≥32	≥16	≥32	≥16	≥2	≤0.25	≤0.25	≥128/4	≥8	≥4	≤2	>4/76	≤0.5	4	–
C18	*kpn*	KPC-2	11	≥64	≥32	≥16	≥32	≥16	≥2	≥16	≥16	≥128/4	≥8	≥4	>32	>4/76	1	≤2	2
C19	*kpn*	KPC-2	11	≥64	≥32	≥16	≥32	≥16	≥2	≥16	≥16	≥128/4	≥8	≥4	>256	>4/76	3	0.5	2
C20	*kpn*	KPC-2	11	≥64	≥32	≥16	≥32	≥16	≥2	≥16	≥16	≥128/4	≥8	≥4	≥64	2/38	≤0.5	4	1
C21	*kpn*	KPC-2	11	≥64	≥32	≥16	≥32	≥16	≥2	≥16	≤0.25	≥128/4	≥8	≥4	>32	2/38	1	≤2	2
C22	*eco*	NDM-5	156	≥64	≥32	≥16	≥32	≥16	≥2	≥16	≥16	≥128/4	≥8	≥4	≤2	>4/76	≤0.5	≤0.5	–
C23	*kpn*	KPC-2	11	≥64	≥32	≥16	≥32	≥16	≥2	≥16	≥16	≥128/4	≥8	≥4	>32	2/38	1	2	1
C24	*kpn*	KPC-71	11	≥64	≥32	≥16	≥32	≥16	≥2	≤0.25	1	≥128/4	≥8	≥4	≤2	>4/76	≤0.5	2	64
C25	*kpn*	ND	11	≥64	≥32	≥16	≥32	≥16	≥2	≥16	≥16	≥128/4	≥8	≥4	≤2	>4/76	≤0.5	4	2
C26	*kpn*	KPC-2	11	≥64	≥32	≥16	≥32	≥16	≥2	≥16	≥16	≥128/4	≥8	≥4	≥64	>4/76	≤0.5	≤2	2
C27	*eco*	NDM-13	361	≥64	≥32	≥16	≥32	8	≥2	≥16	≥16	≥128/4	1	0.5	≤2	>4/76	2	≤0.5	–
C28	*eco*	ND	744	≥64	≥32	≥16	≥32	≥16	≥2	≤0.25	≥16	≥128/4	≥8	≥4	≤2	>4/76	≤0.5	≤0.5	–
C30	*kpn*	KPC-2	11	≥64	≥32	≥16	≥32	≥16	≥2	≥16	≥16	≥128/4	≥8	≥4	≥64	>4/76	≤0.5	≤0.5	2
C31	*eco*	NDM-5	10	≥64	≥32	≥16	≥32	≥16	≥2	≥16	≥16	≥128/4	≥8	≥4	≤2	>4/76	≤0.5	4	–
C32	*kpn*	KPC-2	11	≥64	≥32	≥16	≥32	≥16	≥2	≥16	≥16	≥128/4	≥8	≥4	≥64	2/38	≤0.5	2	4
C33	*kpn*	KPC-2	11	≥64	≥32	≥16	≥32	≥16	≥2	≥16	≥16	≥128/4	≥8	≥4	≥64	≤1/19	≤0.5	1	1
C34	*ecl*	NDM-1	171	≥64	≥32	≥16	≥32	≥16	≥2	≥16	≥16	≥128/4	≥8	≥4	4	≤1/19	≤0.5	2	–
C35	*kpn*	KPC-2	11	≥64	≥32	≥16	≥32	≥16	≥2	≥16	≥16	≥128/4	≥8	≥4	≥64	>4/76	0.5	≤2	1
C36	*eco*	NDM-5	648	≥64	≥32	≥16	≥32	≥16	≥2	≥16	≥16	≥128/4	≥8	≥4	≤2	>4/76	≤0.5	0.125	–
C37	*ecl*	NDM-1	171	≥64	≥32	≥16	≥32	≥16	≥2	≥16	≥16	≥128/4	≥8	≥4	2	≤1/19	2	≤2	–
C38	*kpn*	KPC-2, NDM-1	11	≥64	≥32	≥16	≥32	≥16	≥2	≥16	≥16	≥128/4	≥8	≥4	0.5	>4/76	1.5	≤2	>256
C39	*kpn*	KPC-2	11	≥64	≥32	≥16	≥32	≥16	≥2	≥16	≥16	≥128/4	≥8	≥4	0.25	≤1/19	2	≤2	2
C40	*kpn*	ND	7	≥64	≥32	≥16	≥32	≥16	≥2	2	4	≥128/4	≥8	≥4	1	>4/76	≤1	4	0.125
C41	*kpn*	KPC-2	11	≥64	≥32	≥16	≥32	≥16	≥2	≥16	≥16	≥128/4	≥8	≥4	≥64	≤1/19	2	2	2
C42	*kpn*	KPC-2	11	≥64	≥32	≥16	≥32	≥16	≥2	≥16	≥16	≥128/4	≥8	≥4	0.5	≤1/19	1	≤1	2
C43	*kpn*	KPC-2	11	≥64	≥32	≥16	≥32	≥16	≥2	≥16	≥16	≥128/4	≥8	≥4	0.5	≤1/19	1.5	0.5	2
C44	*kpn*	KPC-2	11	≥64	≥32	≥16	≥32	≥16	≥2	≥16	≥16	≥128/4	≥8	≥4	≥64	≤1/19	1.5	2	2
C45	*kpn*	KPC-2	11	≥64	≥32	≥16	≥32	≥16	≥2	≥16	≥16	≥128/4	≥8	≥4	≥64	≤1/19	2	≤2	2
C46	*kpn*	KPC-2	11	≥64	≥32	≥16	≥32	≥16	≥2	≥16	≥16	≥128/4	≥8	≥4	0.5	>4/76	≤1	2	1
C47	*kpn*	ND	14	≥64	≥32	≥16	≥32	≥16	≥2	≥16	≥16	≥128/4	≥8	≥4	1	≤1/19	2	2	0.25
C48	*kpn*	KPC-2, NDM-1	11	≥64	≥32	≥16	≥32	≥16	≥2	≥16	≥16	≥128/4	≥8	≥4	0.5	>4/76	≤1	2	>256
C49	*kpn*	KPC-2	11	≥64	≥32	≥16	≥32	≥16	≥2	≥16	≥16	≥128/4	≥8	≥4	≥64	≤1/19	≤1	2	2
C50	*kpn*	KPC-2	11	≥64	≥32	≥16	≥32	≥16	≥2	≥16	≥16	≥128/4	≥8	≥4	≥64	≤1/19	1	2	2
C51	*kpn*	KPC-2	11	≥64	≥32	≥16	≥32	≥16	≥2	≥16	≥16	≥128/4	≥8	≥4	≤2	>4/76	≤0.5	2	2
C52	*kpn*	KPC-2	11	≥64	≥32	≥16	≥32	≥16	≥2	≥16	≥16	≥128/4	≥8	≥4	≤2	>4/76	2	2	2
C54	*kpn*	KPC-2	11	≥64	≥32	≥16	≥32	≥16	≥2	≥16	≥16	≥128/4	≥8	≥4	≥64	>4/76	2	≤2	2
C55	*kpn*	KPC-2	11	≥64	≥32	≥16	≥32	≥16	≥2	≥16	≥16	≥128/4	≥8	≥4	≥64	≤1/19	1	≤2	2
C56	*eco*	NDM-5	2325	≥64	≥32	≥16	≥32	≥16	≥2	≥16	≥16	≥128/4	≥8	≥4	≥64	>4/76	0.25	≤2	–
C57	*kpn*	KPC-2	11	≥64	≥32	≥16	≥32	≥16	≥2	≥16	≥16	≥128/4	≥8	≥4	≥64	>4/76	2	≤2	8
C58	*kae*	ND	ND	≥64	≥32	≥16	≥32	≥16	≥2	≥16	≥16	≥128/4	≤0.12	≤0.25	4	≤1/19	≤0.5	≤2	4
C59	*ecl*	NDM-1	171	≥64	≥32	≥16	≥32	≥16	≥2	≥16	≥16	≥128/4	≥8	≥4	2	≤1/19	1.5	≤2	–
C60	*kpn*	NDM-1	3948	≥64	≥32	≥16	≥32	≥16	≥2	≥16	≥16	≥128/4	≥8	≥4	0.5	>4/76	2	1	–
C61	*kpn*	KPC-2	11	≥64	≥32	≥16	≥32	≥16	≥2	≥16	≥16	≥128/4	≥8	≥4	≤2	>4/76	≤0.5	4	2
C62	*cfr*	ND	ND	≥64	≥32	≥16	≥32	≥16	≥2	≥16	≥16	≥128/4	≤0.12	≤0.25	≤2	≤1/19	≤0.5	≤0.5	0.125
C63	*kpn*	KPC-2, NDM-1	11	≥64	≥32	≥16	≥32	≥16	≥2	≥16	≥16	≥128/4	≥8	≥4	≥64	>4/76	≤0.5	4	>256
C64	*kpn*	KPC-2	11	≥64	≥32	≥16	≥32	≥16	≥2	≥16	≥16	≥128/4	≥8	≥4	≥64	≤1/19	≤0.5	4	2
C65	*kpn*	KPC-2	11	≥64	≥32	≥16	≥32	≥16	≥2	≥16	≥16	≥128/4	≥8	≥4	≥64	>4/76	≤0.5	4	1
C66	*eco*	NDM-13	ND	≥64	≥32	≥16	≥32	≤4	≥2	≥16	≥16	≥128/4	≤0.12	≤0.25	≤2	≤1/19	≤0.5	≤0.5	–

Gray-shaded were interpreted as sensitive; SCF cefoperazone/sulbactam, CROceftriaxone, CAZ ceftazidime, FEP cefepime, ATM aztreonam, ETP ertapenem, IPM imipenem, MEM meropenem, TZP piperacillin/tazobactam, LEV levofloxacin, CIP ciprofloxacin, AK amikacin, SXT sulfamethoxazole-trimethoprim, TGC tigecycline, COL polymyxin B, CZA ceftazidime/avibactam, kpn *Klebsiella pneumoniae, eco Escherichia coli, ecl Enterobacter cloacae, kae Klebsiella aerogenes, cfr* C*itrobacter freundii;*KPC-71, a variant of KPC-2, was detected in one isolate (C24); -, not determined (CZA not tested against NDM-producing isolates due to intrinsic resistance); ND, not detected (Carbapenemase column) or not determined (ST column).

### 3.4 Phylogenetic tree of 48 *Klebsiella pneumoniae* strains

**Figure 2:**
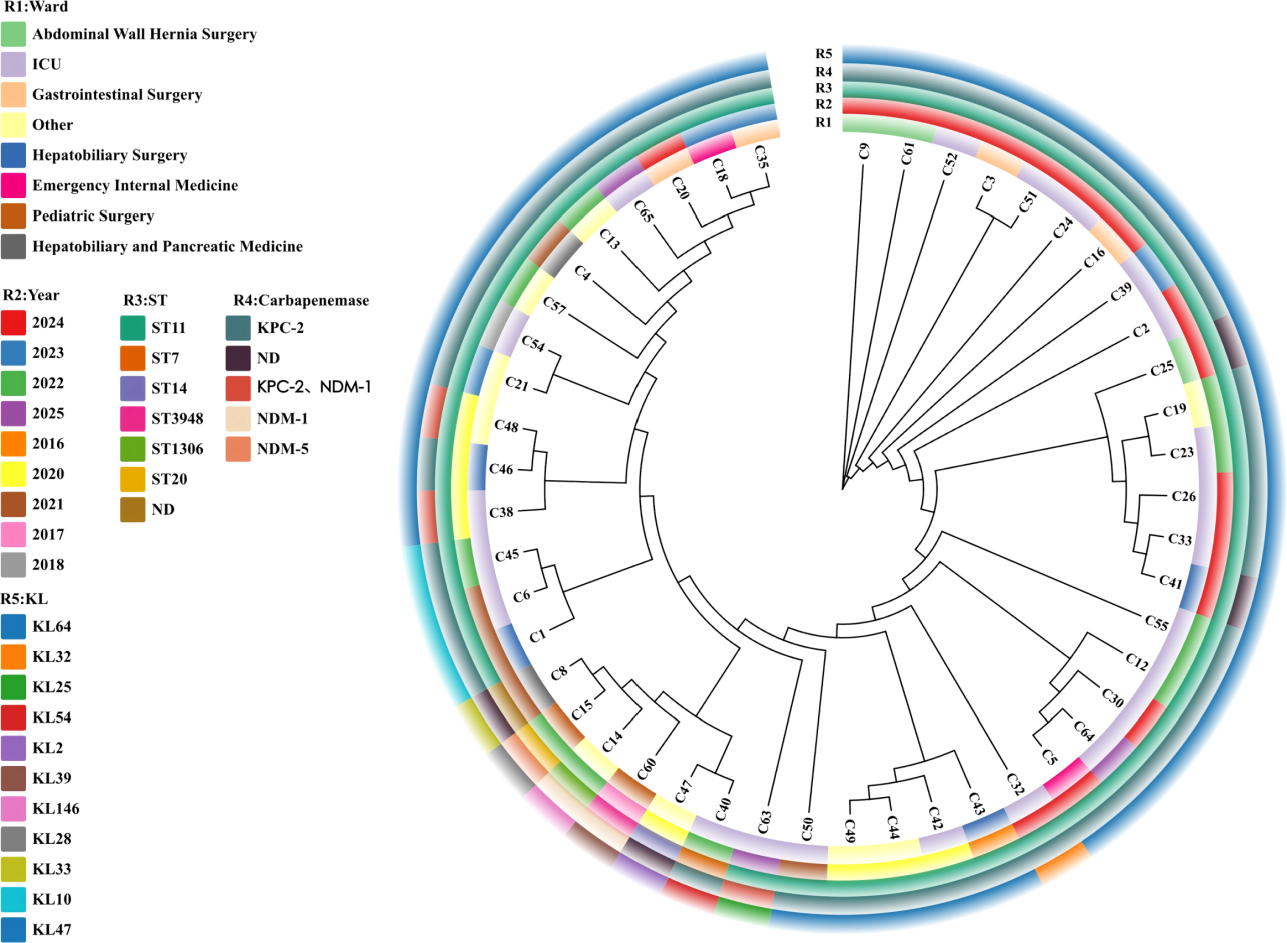
Core genome SNP phylogeny of CRKP. Midpoint-rooted core genome SNP phylogeny of 48 K*. pneumoniae* isolates. Rings indicate ward (R1), year (R2), sequence type (R3), carbapenemase genes (R4) and capsule locus type (R5).

The phylogenetic tree constructed based on core genome SNPs revealed that ST11 was the most common sequence type (87.5%, 42/48), forming a distinct phylogenetic clade, whereas other isolates belonged to sporadic STs including ST3948, ST1306, ST7, ST14 and ST20. Genetic relatedness among ST11 isolates was analyzed using a ≤21 SNPs cutoff value, and five major genetic clades were identified (**Figure 2**). Clade A contained eight isolates (C3, C9, C16, C24, C39, C51, C52, C61) with pairwise SNP differences of 1–21, representing the largest phylogenetic clade. Core isolates in this clade (C3, C9, C16, C24, C52, C61) exhibited extremely close genetic homology (1–4 SNPs), whereas peripheral isolates (C39, C51) differed from core strains by 15–21 SNPs. These strains were mainly collected from ICU and gastrointestinal surgery wards between 2023 and 2024. Clade B included three isolates (C38, C46, C48) with 8–10 SNP differences, recovered from ICU and hepatobiliary surgery in 2020. Of note, C38 and C48 co-carried *bla*_KPC-2_ and *bla*_NDM-1_, while C46 harbored only *bla*_KPC-2_. Clade C consisted of three isolates (C19, C23, C25) with 2–13 SNP differences, among which C19 and C23 differed by merely 2 SNPs. Clade D comprised three isolates (C1, C6, C45) with 5–9 SNP differences, all carrying *bla*_KPC-2_. Clade E contained seven isolates (C4, C13, C18, C20, C35, C57, C65) with 6–19 SNP differences. All strains in this clade carried *bla*_KPC-2_ and were isolated from multiple departments during 2021–2025. The remaining 24 isolates showed >21 SNP differences from these major clades. Regarding capsule locus (KL) types, ST11 isolates were primarily associated with KL64 and KL47 (**Figure 2**; **Supplementary Table 3**).

### 3.5 Major virulence genes and plasmid replicons in 48 *Klebsiella pneumoniae* strains

**Figure 3:**
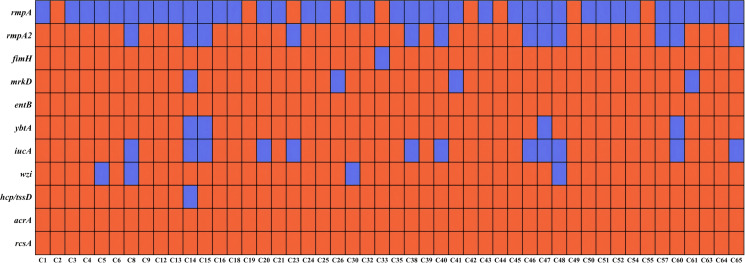
Distribution of virulence-associated genes among 48 CRKP isolates. Red indicates the presence of the gene; Blue indicates its absence.

The carriage characteristics of core virulence genes among the 48 K*. pneumoniae* strains showed significant differences (**Figure 3**). The carriage rate of the hypervirulence marker gene *rmpA2* was 75.0% (36/48), while that of *rmpA* was 18.8% (9/48). All *rmpA*-positive strains were co-positive for both *rmpA* and *rmpA2*, with no strains carrying *rmpA* alone. The carriage rates of adhesion-related genes *fimH* and *mrkD* were 97.9% (47/48) and 91.7% (44/48), respectively. The carriage rates of iron acquisition system genes *entB*, *ybtA*, and *iucA* were 100% (48/48), 91.7% (44/48), and 75.0% (36/48), respectively, with a 100% co-occurrence rate between *ybtA* and *rmpA2*. The carriage rates of capsular synthesis gene *wzi* and type VI secretion system core gene *hcp/tssD* were 91.7% (44/48) and 97.9% (47/48), respectively. The carriage rates of efflux pump gene *acrA* and virulence regulatory gene *rcsA* were both 100% (48/48) (**Figure 3**).

**Figure 4:**
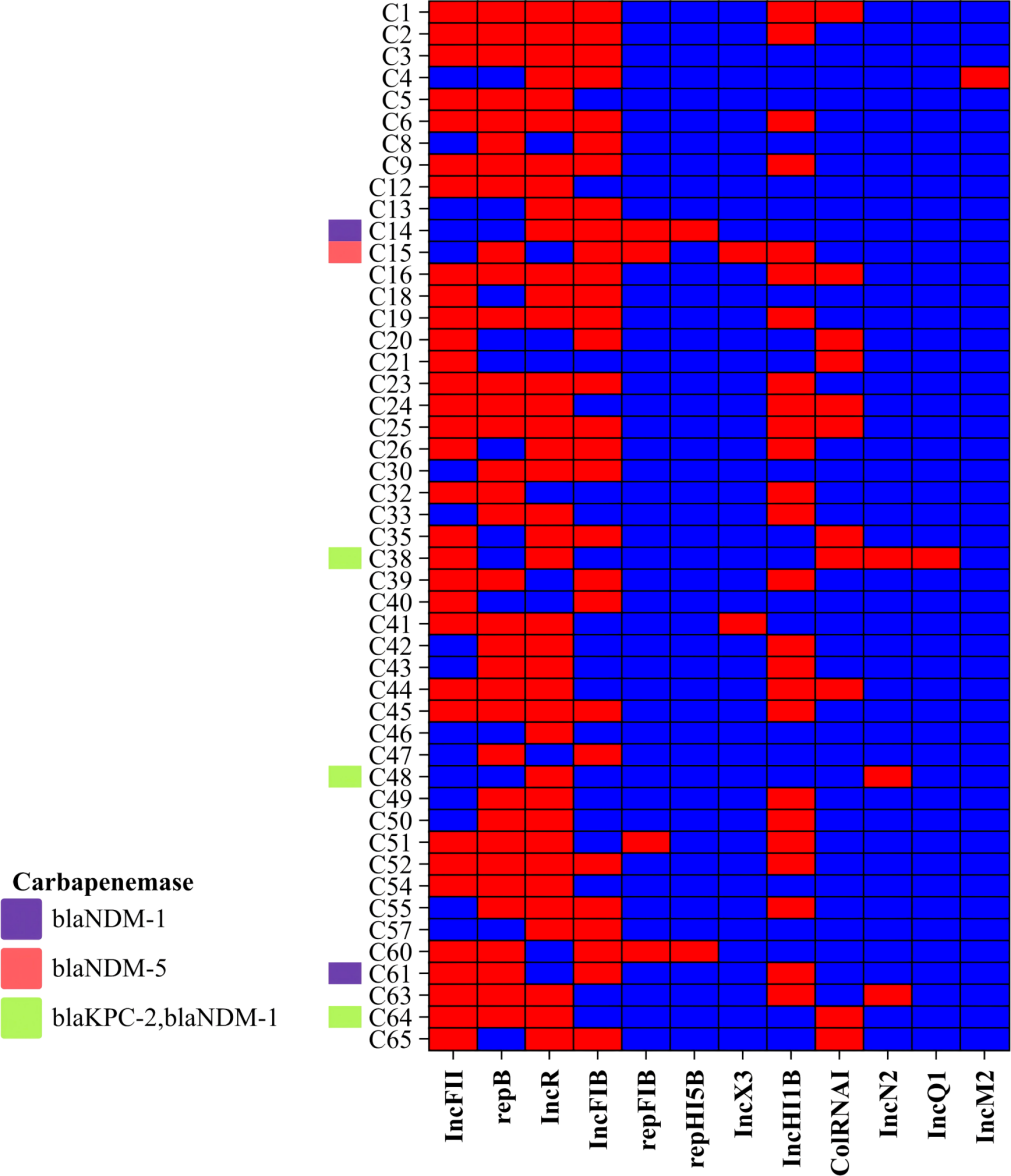
Heat-map displaying plasmid replicon profiles of 48 CRKP isolates. Red indicates the presence of the gene; Blue indicates its absence. The left sidebar indicates carbapenemase type: purple, *bla*_NDM-1_; red, *bla*_NDM-5_; green, *bla*_KPC-2_+*bla*_NDM-1_.

Among the 48 CRKP strains, the predominant replicons were IncR (79.2%, 38/48), *repB* (70.8%, 34/48), IncFII (66.7%, 32/48), IncFIB (60.4%, 29/48), and IncHI1B (52.1%, 25/48). Three dual-enzyme strains (6.3%) (C38, C48, C63) carried IncFII/IncN (**Figure 4**).

## 4 Discussion

CRE has been widely disseminated worldwide, with 30-day mortality rates reaching 30% in CRKP-infected patients (Ma et al., 2023). In this study, CRE-infected patients were predominantly elderly males (mean age 64.6 years, 74.2% ≥60 years), consistent with previous reports identifying this demographic as high-risk (Zhang et al., 2018; Grundmann et al., 2017) ICU admission (40.3% of isolates), recent surgery (77.4% of patients), and invasive procedures (75.8%) were major risk factors, supporting targeted surveillance in high-risk populations (Falcone et al., 2023). Prior carbapenem exposure was common (53.2%), consistent with antimicrobial pressure as a recognized risk factor for CRE selection, underscoring the need for ongoing antimicrobial stewardship (Tamma et al., 2022; Bonomo et al., 2018). The main specimen sources were ascites (50.0%), pleural fluid, and bile (19.4% each), indicating that abdominal and thoracic cavities are high-incidence sites for invasive CRE infections due to complex anatomy and poor drainage (Wang et al., 2022; Shafiekhani et al., 2025).

*K. pneumoniae* was the dominant species (77.4%), and ST11 accounted for 87.5% of CRKP isolates, carrying *bla*_KPC-2_ and predominantly corresponding to KL64 and KL47 capsule types. These molecular features are consistent with reports from most regions in China (Lei et al., 2024; Wang et al., 2018; Ernst et al., 2020; Pu et al., 2023) but differ from the ST258/ST512 lineages prevalent in North America and Europe (Ma et al., 2023; Mathers et al., 2015; Castanheira et al., 2013) and the ST512 lineage associated with KPC-3 in Italy (Arcari et al., 2023). Phylogenetic analysis resolved ST11 isolates into five clonal clusters (Clade A-E) using a ≤21 SNP cutoff, consistent with established criteria for detecting nosocomial transmission of ST11-KL64 *K. pneumoniae* (Wang et al., 2023). The extremely tight clustering within Clade A (1-4 SNPs) and its concentration in ICU and gastrointestinal surgery during 2023-2024 is compatible with recent intra-hospital spread, as observed in prior outbreak investigations (Zeng et al., 2021). Similarly, Clade B (8-10 SNPs), recovered from ICU and hepatobiliary surgery in 2020, included dual-carbapenemase isolates, raising concern for clonal dissemination of difficult-to-treat strains. In contrast, Clade E showed broader spatiotemporal distribution (6-19 SNPs) across multiple departments during 2021-2025, suggesting persistent endemic circulation rather than a point-source outbreak. The remaining isolates (>21 SNPs) likely represent sporadic introductions. Although core-genome SNP analysis alone cannot prove direct nosocomial transmission without accompanying plasmid sequencing, the spatiotemporal overlap of tight clusters supports the value of WGS for targeted surveillance in high-risk departments.

All *E. cloacae* isolates belonged to ST171 and carried *bla*_NDM-1_ with 100% AmpC and ESBL positivity, forming a stable resistant genotype, consistent with reports that ST171 represents a high−risk global clone (Gomez-Simmonds et al., 2018). In contrast, *E. coli* showed high genetic diversity without a predominant lineage, indicating limited clonal spread, consistent with reports of plasmid-mediated resistance in genetically diverse *E. coli* populations (Li et al., 2024). *bla*_KPC-2_ was the dominant carbapenemase gene in CRKP, while *bla*_NDM_ variants were more common in *E. coli* and *E. cloacae*, reflecting species-specific resistance mechanisms (Adelman et al., 2022; Bonomo et al., 2018). Several ST11 isolates co-harbored *bla*_KPC-2_ and *bla*_NDM-1_, leading to resistance to ceftazidime-avibactam. All isolates were multidrug-resistant but remained largely susceptible to tigecycline, polymyxin B, and ceftazidime-avibactam (except NDM-producing strains), suggesting retained *in vitro* activity, consistent with reported susceptibility patterns in CRE isolates (Durante-Mangoni et al., 2019). The low rate of mcr genes indicates that polymyxins remain active in this region. Virulence analysis showed that 75.0% of CRKP isolates carried *rmpA2*, consistent with the typical molecular characteristics of hvKp (Tan et al., 2019; Russo and Marr, 2019). High carriage rates of iron uptake and adhesion genes further enhance pathogenicity, as reported previously (Ernst et al., 2020). Most fatal cases were caused by CRKP, consistent with the high invasiveness and poor prognosis of hv−CRKP (Pu et al., 2023; Wang et al., 2022; Lei et al., 2024). CRE infections from sterile body fluids are associated with high mortality (30–50%) and represent a critical high-risk population worthy of focused study. These findings provide baseline molecular epidemiologic data for this understudied infection type in regions where ST11-KPC-2 is endemic.

## 5 Limitations

This single-center retrospective study (n=62) lacked contemporaneous non-sterile site controls, preventing determination of whether ST11 predominance reflects specific invasive pathogenicity or high background abundance. SNP-based phylogenetic clustering (≤21 SNPs) suggests close genetic relatedness among some isolates but cannot confirm direct nosocomial transmission without further plasmid sequencing. Incomplete clinical data may also limit causal inference for risk factors.

## 6 Conclusion

This study described the molecular epidemiology and clinical characteristics of CRE from sterile body fluids over a 10-year period at a tertiary hospital. ST11 *K. pneumoniae* carrying *bla*_KPC-2_ was the most prevalent genotype, accompanied by *rmpA2* detected in 75.0% of *K. pneumoniae* isolates. *E. cloacae* isolates belonged to ST171 with consistent resistance genotypes, whereas *E. coli* exhibited high genetic diversity. The main carbapenemase genes were *bla*_KPC-2_ and *bla*_NDM_ variants, and tigecycline, polymyxin B, and ceftazidime-avibactam remained active against most isolates. ICU admission was significantly associated with mortality, and most patients had prior histories of surgery or invasive procedures. In settings where ST11-KPC-2 is endemic, surveillance of clonal transmission in high-risk departments may inform local infection control strategies.

## Data Availability

The data presented in the study are deposited in the NCBI repository, BioProject accession number PRJNA1430231. The raw sequencing reads and genome assemblies corresponding to individual BioSample accession numbers are listed in **Supplementary Table S1**.
