# Supplementary material for: Carbapenem-resistant enterobacterales in sterile body fluids: ten-year population genomics and clinical risk factors in a tertiary hospital, 2016-2025
Source: Front Cell Infect Microbiol. 2026 Jun 2;16:1821740. doi: 10.3389/fcimb.2026.1821740 (PMC13269256; doi:10.3389/fcimb.2026.1821740)
Supplement: Supplementary file 1 [file Table1.doc]

Table S1. BioSample accession numbers and final species designations of the 62 CRE isolates.

| **Strain ID** | **BioSample Accession** | **Organism** |
| --- | --- | --- |
| **C1** | SAMN56275064 | *kpn* |
| **C2** | SAMN56275065 | *kpn* |
| **C3** | SAMN56275066 | *kpn* |
| **C4** | SAMN56275067 | *kpn* |
| **C5** | SAMN56275068 | *kpn* |
| **C6** | SAMN56275069 | *kpn* |
| **C8** | SAMN56275070 | *kpn* |
| **C9** | SAMN56275071 | *kpn* |
| **C11** | SAMN56275072 | *ecl* |
| **C12** | SAMN56275073 | *kpn* |
| **C13** | SAMN56275074 | *kpn* |
| **C14** | SAMN56275075 | *kpn* |
| **C15** | SAMN56275076 | *kpn* |
| **C16** | SAMN56275077 | *kpn* |
| **C17** | SAMN56275078 | *eco* |
| **C18** | SAMN56275079 | *kpn* |
| **C19** | SAMN56275080 | *kpn* |
| **C20** | SAMN56275081 | *kpn* |
| **C21** | SAMN56275082 | *kpn* |
| **C22** | SAMN56275083 | *eco* |
| **C23** | SAMN56275084 | *kpn* |
| **C24** | SAMN56275085 | *kpn* |
| **C25** | SAMN56275086 | *kpn* |
| **C26** | SAMN56275087 | *kpn* |
| **C27** | SAMN56275088 | *eco* |
| **C28** | SAMN56275089 | *eco* |
| **C30** | SAMN56275090 | *kpn* |
| **C31** | SAMN56275091 | *eco* |
| **C32** | SAMN56275092 | *kpn* |
| **C33** | SAMN56275093 | *kpn* |
| **C34** | SAMN56275094 | *ecl* |
| **C35** | SAMN56275095 | *kpn* |
| **C36** | SAMN56275096 | *eco* |
| **C37** | SAMN56275097 | *ecl* |
| **C38** | SAMN56275098 | *kpn* |
| **C39** | SAMN56275099 | *kpn* |
| **C40** | SAMN56275100 | *kpn* |
| **C41** | SAMN56275101 | *kpn* |
| **C42** | SAMN56275102 | *kpn* |
| **C43** | SAMN56275103 | *kpn* |
| **C44** | SAMN56275104 | *kpn* |
| **C45** | SAMN56275105 | *kpn* |
| **C46** | SAMN56275106 | *kpn* |
| **C47** | SAMN56275107 | *kpn* |
| **C48** | SAMN56275108 | *kpn* |
| **C49** | SAMN56275109 | *kpn* |
| **C50** | SAMN56275110 | *kpn* |
| **C51** | SAMN56275111 | *kpn* |
| **C52** | SAMN56275112 | *kpn* |
| **C54** | SAMN56275113 | *kpn* |
| **C55** | SAMN56275114 | *kpn* |
| **C56** | SAMN56275115 | *eco* |
| **C57** | SAMN56275116 | *kpn* |
| **C58** | SAMN56275117 | *kae* |
| **C59** | SAMN56275118 | *ecl* |
| **C60** | SAMN56275119 | *kpn* |
| **C61** | SAMN56275120 | *kpn* |
| **C62** | SAMN56275121 | *cfr* |
| **C63** | SAMN56275122 | *kpn* |
| **C64** | SAMN56275123 | *kpn* |
| **C65** | SAMN56275124 | *kpn* |
| **C66** | SAMN56275125 | *eco* |

*kpn* *Klebsiella pneumoniae, eco Escherichia coli, ecl Enterobacter cloacae, kae Klebsiella aerogenes, cfr* C*itrobacter freundii*
