# Supplementary material for: Carbapenem-resistant enterobacterales in sterile body fluids: ten-year population genomics and clinical risk factors in a tertiary hospital, 2016-2025
Source: Front Cell Infect Microbiol. 2026 Jun 2;16:1821740. doi: 10.3389/fcimb.2026.1821740 (PMC13269256; doi:10.3389/fcimb.2026.1821740)
Supplement: Supplementary file 2 [file Table2.doc]

Supplementary Table S2. Clinical and epidemiological data of patients with CRE isolated from sterile body fluids (n = 62).

| **Patient ID** | **Strain** | **Age** | **Sex** | **Ward** | **Year** | **Length of Stay  (d)** | **ICU admission in the past 12 months** | **History of surgery in the past 6 months** | **Source** | **Invasive Procedures** | **Antimicrobial Exposure Prior to CRE Detection** | **Outcome** |
| --- | --- | --- | --- | --- | --- | --- | --- | --- | --- | --- | --- | --- |
| **C1** | kpn | 66 | F | Hepatobiliary Surgery | 2021 | 9 | Yes | Yes | Ascites | NO | TZP | Survived |
| **C2** | kpn | 86 | M | ICU | 2024 | 13 | Yes | Yes | Bile | UC | MEM | Survived |
| **C3** | kpn | 71 | F | Gastrointestinal Surgery | 2024 | 48 | No | Yes | Ascites | UC、CVC | CXM、MEM、SCF | Survived |
| **C4** | kpn | 76 | F | Hepatobiliary and Pancreatic Medicine | 2021 | 29 | Yes | Yes | Bile | UC、CVC | MEM、FOX | Survived |
| **C5** | kpn | 78 | M | Emergency Internal Medicine | 2024 | 34 | No | No | Nephrostomy Fluid | UC、MV | MEM、SCF | Survived |
| **C6** | kpn | 54 | M | ICU | 2021 | 81 | Yes | Yes | CSF | UC、CVC、MV | MEM、TGC | Survived |
| **C8** | kpn | 72 | F | Hepatobiliary and Pancreatic Medicine | 2021 | 5 | No | Yes | Bile | NO | SCF | Survived |
| **C9** | kpn | 53 | M | Abdominal Wall Hernia Surgery | 2024 | 22 | No | Yes | Subphrenic Fluid | UC、CVC | CXM、SCFMEM | Survived |
| **C11** | ecl | 68 | F | Gastroenterology | 2022 | 151 | No | Yes | Ascites | NO | SCF、TGC | Survived |
| **C12** | kpn | 77 | M | ICU | 2022 | 106 | Yes | Yes | Pleural effusion | CVC、MV | PB | Died |
| **C13** | kpn | 87 | F | Geriatric Respiratory Medicine | 2022 | 10 | Yes | No | Pleural effusion | UC | TZP | Died |
| **C14** | kpn | 55 | F | Gastroenterology | 2022 | 14 | No | Yes | Pleural effusion | NO | CXM | Survived |
| **C15** | kpn | 5 | F | Pediatric Surgery | 2022 | 28 | No | Yes | Ascites | UC、MV | MEZ、CRO、MEM | Survived |
| **C16** | kpn | 71 | F | Gastrointestinal Surgery | 2024 | 48 | Yes | Yes | Ascites | NO | Unobtainable | Survived |
| **C17** | eco | 66 | M | Gastrointestinal Surgery | 2024 | 39 | No | Yes | Ascites | UC、CVC、MV | SCF、CXM | Survived |
| **C18** | kpn | 81 | M | Emergency Internal Medicine | 2023 | 15 | Yes | No | Ascites | UC | MEM、MXF | Survived |
| **C19** | kpn | 72 | F | General Surgery | 2022 | 33 | Yes | Yes | Pleural effusion | BD | TZP、LEV | Died |
| **C20** | kpn | 84 | M | Gastrointestinal Surgery | 2024 | 19 | No | Yes | Bile | UC、MV | SCF、IPM | Survived |
| **C21** | kpn | 72 | M | Gastroenterology | 2023 | 64 | No | Yes | Ascites | CVC | CZA、TGC | Survived |
| **C22** | eco | 75 | M | Colorectal and Anal Surgery | 2024 | 18 | No | Yes | Pelvic Effusion | UC、CVC | MXF、CMZ | Survived |
| **C23** | kpn | 61 | F | ICU | 2022 | 67 | Yes | Yes | Ascites | UC、CVC、MV | TZP、MEM、SCF、TGC | Survived |
| **C24** | kpn | 74 | M | ICU | 2024 | 151 | Yes | Yes | Ascites | UC、CVC、MV | CXM、TZP、MEM、CZA、AK | Survived |
| **C25** | kpn | 53 | M | Abdominal Wall Hernia Surgery | 2024 | 22 | No | Yes | Subphrenic Fluid | UC、CVC | CXM、SCF、MEM | Survived |
| **C26** | kpn | 70 | F | ICU | 2024 | 21 | Yes | Yes | Ascites | UC、CVC、MV | TZP、 | Survived |
| **C27** | eco | 70 | F | Gastroenterology | 2024 | 4 | No | Yes | Bile | NO | None | Survived |
| **C28** | eco | 61 | F | Hepatobiliary and Pancreatic Medicine | 2024 | 8 | No | Yes | Bile | NO | SCF | Survived |
| **C30** | kpn | 96 | M | ICU | 2024 | 25 | Yes | Yes | Bile | UC、CVC、MV | TZP、LEV | Died |
| **C31** | eco | 67 | M | ICU | 2024 | 12 | Yes | No | Ascites | UC、CVC | CMZ、MEM | Died |
| **C32** | kpn | 86 | M | ICU | 2024 | 18 | Yes | No | Ascites | UC | CZA | Died |
| **C33** | kpn | 96 | M | ICU | 2024 | 25 | Yes | Yes | Pleural effusion | UC、CVC、MV | TZP、LEV、MEM | Died |
| **C34** | ecl | 73 | M | ICU | 2024 | 3 | Yes | No | Pleural effusion | UC、CVC、MV | MEM | Died |
| **C35** | kpn | 60 | M | Gastrointestinal Surgery | 2023 | 30 | No | No | Ascites | NO | SCF、CAZ、 | Survived |
| **C36** | eco | 20 | F | Gastrointestinal Surgery | 2024 | 6 | No | Yes | Ascites | NO | SCF | Survived |
| **C37** | ecl | 50 | M | ICU | 2019 | 35 | Yes | Yes | Ascites | UC、CVC、MV | TZP、ETM、MEZ、IPM | Survived |
| **C38** | kpn | 56 | M | ICU | 2020 | 16 | Yes | Yes | Ascites | UC、CVC、MV | MEM、TGC | Died |
| **C39** | kpn | 82 | F | ICU | 2023 | 22 | Yes | No | Bile | UC | MEM | Survived |
| **C40** | kpn | 84 | M | ICU | 2020 | 159 | Yes | Yes | Perihepatic Fluid | UC、CVC、MV | IPM | Survived |
| **C41** | kpn | 73 | M | Hepatobiliary Surgery | 2023 | 25 | Yes | Yes | Bile | UC、CVC | CXM | Survived |
| **C42** | kpn | 52 | M | ICU | 2020 | 11 | Yes | No | Pleural effusion | UC | NO | Survived |
| **C43** | kpn | 67 | F | Hepatobiliary Surgery | 2016 | 3 | No | Yes | Ascites | UC | CRO | Survived |
| **C44** | kpn | 7 | F | Pediatric Internal Medicine | 2020 | 710 | No | Yes | Pleural effusion | UC、CVC、MV | CTX、MEM | Survived |
| **C45** | kpn | 72 | M | ICU | 2022 | 35 | Yes | No | Ascites | UC、CVC、MV | SCF、MEM | Died |
| **C46** | kpn | 70 | M | Hepatobiliary Surgery | 2020 | 42 | No | Yes | Ascites | UC、CVC、MV | TZP、IPM、MXF | Survived |
| **C47** | kpn | 59 | M | Nephrology | 2020 | 82 | Yes | Yes | Ascites | UC、CVC、MV | CAZ、MEM | Survived |
| **C48** | kpn | 61 | M | Nephrology | 2020 | 23 | Yes | No | Pleural effusion | UC、CVC、MV | LEV、MEM | Survived |
| **C49** | kpn | 0 | F | Neonatology | 2020 | 45 | Yes | No | CSF | NO | CAZ、MEM | Survived |
| **C50** | kpn | 83 | M | ICU | 2021 | 27 | Yes | Yes | Pleural effusion | CVC | TZP、MEM | Survived |
| **C51** | kpn | 66 | M | ICU | 2024 | 54 | Yes | Yes | Ascites | UC、CVC、MV | MEM | Survived |
| **C52** | kpn | 80 | F | ICU | 2024 | 38 | Yes | Yes | Ascites | UC、CVC | CXM、SCF、IPM、TGC、 | Died |
| **C54** | kpn | 61 | M | ICU | 2018 | 36 | Yes | Yes | Bile | MV | CAZ、MEM、IPM | Survived |
| **C55** | kpn | 74 | M | ICU | 2022 | 49 | Yes | Yes | Pleural effusion | NO | CXM | Survived |
| **C56** | eco | 63 | M | General Surgery | 2018 | 61 | No | Yes | Ascites | UC、CVC | FOX、SCF | Survived |
| **C57** | kpn | 80 | F | Chinese Medicine Department | 2022 | 42 | No | Yes | Pleural effusion | UC | NO | Survived |
| **C58** | kae | 65 | M | Gastrointestinal Surgery | 2022 | 32 | No | Yes | Ascites | UC、CVC | CXM、TZP、IPM、 | Survived |
| **C59** | ecl | 70 | M | Gastrointestinal Surgery | 2022 | 30 | No | Yes | Ascites | NO | TZP | Survived |
| **C60** | kpn | 13 | M | Pediatric Surgery | 2017 | 30 | No | Yes | Ascites | UC | CAZ | Survived |
| **C61** | kpn | 39 | M | Abdominal Wall Hernia Surgery | 2024 | 20 | Yes | Yes | Ascites | CVC | CXM | Survived |
| **C62** | cfr | 65 | M | Hepatobiliary and Pancreatic Medicine | 2025 | 10 | No | Yes | Bile | NO | SCF | Survived |
| **C63** | kpn | 44 | M | ICU | 2025 | 19 | Yes | No | Ascites | CVC | SCF、MEM | Died |
| **C64** | kpn | 71 | M | ICU | 2025 | 55 | Yes | No | Ascites | UC、CVC、MV | LEV、MEM | Survived |
| **C65** | kpn | 83 | M | ICU | 2025 | 42 | Yes | Yes | Ascites | UC、CVC、MV | MEM、TGC | Survived |
| **C66** | eco | 65 | M | Hepatobiliary and Pancreatic Medicine | 2025 | 10 | No | Yes | Bile | NO | SCF | Survived |

CRE, Carbapenem-Resistant Enterobacterales; ICU, intensive care unit; LOS, length of stay; SCF, cefoperazone–sulbactam; UC, urinary catheter; CVC, central venous catheter; MV, mechanical ventilation; AK, amikacin; AMC, amoxicillin -clavulanate；ATM, aztreonam; CAZ, ceftazidime; CFIX，cefixime； CMZ，cefmetazole；CRE, Carbapenem-Resistant Enterobacterales; CRO, ceftriaxone； CTX, cefotaxime；CXM, cefuroxime; CZA, ceftazidime–avibactam; ETM, etimicin; FOX, cefoxitin; IPM, imipenem； LEV, levofloxacin; MEM, meropenem; MEZ, mezlocillin；MXF,moxifloxacin; MV, mechanical ventilation; PB, polymyxin B; SCF, cefoperazone–sulbactam; TGC, tigecycline; TZP, piperacillin–tazobactam.
