# Supplementary material for: Carbapenem-resistant enterobacterales in sterile body fluids: ten-year population genomics and clinical risk factors in a tertiary hospital, 2016-2025
Source: Front Cell Infect Microbiol. 2026 Jun 2;16:1821740. doi: 10.3389/fcimb.2026.1821740 (PMC13269256; doi:10.3389/fcimb.2026.1821740)
Supplement: Supplementary file 3 [file Table3.doc]

Supplementary Table S3. Pairwise core genome SNP differences among CRKP isolates within clonal clusters (Clade A-E).

Clade A

| Isolates | C3 | C9 | C16 | C24 | C51 | C52 | C61 | C39 |
| --- | --- | --- | --- | --- | --- | --- | --- | --- |
| C3 | 0 | 2 | 2 | 3 | 4 | 1 | 2 | 17 |
| C9 | 2 | 0 | 4 | 3 | 6 | 1 | 2 | 19 |
| C16 | 2 | 4 | 0 | 1 | 6 | 3 | 4 | 15 |
| C24 | 3 | 3 | 1 | 0 | 7 | 2 | 3 | 16 |
| C51 | 4 | 6 | 6 | 7 | 0 | 5 | 6 | 21 |
| C52 | 1 | 1 | 3 | 2 | 5 | 0 | 1 | 18 |
| C61 | 2 | 2 | 4 | 3 | 6 | 1 | 0 | 19 |
| C39 | 17 | 19 | 15 | 16 | 21 | 18 | 19 | 0 |

Clade B

| Isolates | C38 | C46 | C48 |
| --- | --- | --- | --- |
| C38 | 0 | 8 | 10 |
| C46 | 8 | 0 | 10 |
| C48 | 10 | 10 | 0 |

Clade C

| Isolates | C19 | C23 | C25 |
| --- | --- | --- | --- |
| C19 | 0 | 2 | 11 |
| C23 | 2 | 0 | 13 |
| C25 | 11 | 13 | 0 |

Clade D

| Isolates | C1 | C6 | C45 |
| --- | --- | --- | --- |
| C1 | 0 | 5 | 8 |
| C6 | 5 | 0 | 9 |
| C45 | 8 | 9 | 0 |

Clade E

| Isolates | C35 | C18 | C20 | C65 | C13 | C4 | C57 |
| --- | --- | --- | --- | --- | --- | --- | --- |
| C35 | 0 | 6 | 12 | 12 | 13 | 9 | 15 |
| C18 | 6 | 0 | 10 | 10 | 7 | 7 | 13 |
| C20 | 12 | 10 | 0 | 12 | 13 | 13 | 19 |
| C65 | 12 | 10 | 12 | 0 | 9 | 11 | 11 |
| C13 | 13 | 7 | 13 | 9 | 0 | 8 | 10 |
| C4 | 9 | 7 | 13 | 11 | 8 | 0 | 6 |
| C57 | 15 | 13 | 19 | 11 | 10 | 6 | 0 |

Pairwise core genome SNP differences were calculated using CSI Phylogeny 1.4. Values represent the number of SNP differences between isolates. Isolates differing by ≤21 SNPs were defined as belonging to the same clonal cluster.
